# Western Diet-Fed ApoE Knockout Male Mice as an Experimental Model of Non-Alcoholic Steatohepatitis

**DOI:** 10.3390/cimb44100320

**Published:** 2022-10-08

**Authors:** Felipe N. Camargo, Sandro L. Matos, Layanne C. C. Araujo, Carla R. O. Carvalho, Andressa G. Amaral, João Paulo Camporez

**Affiliations:** 1 Department of Physiology and Biophysics, Institute of Biomedical Sciences, University of Sao Paulo, Av. Bandeirantes nº3900, Ribeirao Preto 14049-900, SP, Brazil; 2 Department of Physiology, Ribeirao Preto Medical School, University of Sao Paulo, Av. Bandeirantes nº3900, Ribeirao Preto 14049-900, SP, Brazil

**Keywords:** NASH, NAFLD, insulin resistance, Western diet

## Abstract

One of the consequences of the Western lifestyle and high-fat diet is non-alcoholic fatty liver disease (NAFLD) and its aggressive form, non-alcoholic steatohepatitis (NASH), which can progress to cirrhosis and hepatocellular carcinoma (HCC) and is rapidly becoming the leading cause of end-stage liver disease or liver transplantation. Currently, rodent NASH models lack significant aspects of the full NASH spectrum, representing a major problem for NASH research. Therefore, this work aimed to characterize a fast rodent model with all characteristic features of NASH. Eight-week-old male ApoE KO mice were fed with Western diet (WD), high fatty diet (HFD) or normal chow (Chow) for 7 weeks. Whole-body fat was increased by ~2 times in WD mice and HFD mice and was associated with increased glucose intolerance, hepatic triglycerides, and plasma ALT and plasma AST compared with Chow mice. WD mice also showed increased galectin-3 expression compared with Chow or HFD mice and increased plasma cholesterol compared with Chow mice. WD and HFD displayed increased hepatic fibrosis and increased F4/80 expression. WD mice also displayed increased levels of plasma MCP-1. Hepatic inflammatory markers were evaluated, and WD mice showed increased levels of TNF-α, MCP-1, IL-6 and IFN-γ. Taken together, these data demonstrated that the ApoE KO mouse fed with WD is a great model for NASH research, once it presents the fundamental parameters of the disease, including hepatic steatosis, fibrosis, inflammation, and metabolic syndrome.

## 1. Introduction

Lifestyle and, more precisely, increased consumption of a high-fat diet largely contribute to the development of obesity, insulin resistance, type 2 diabetes (T2DM), and cardiovascular disease [1]. The set of these abnormalities is called metabolic syndrome, and the central factor in this syndrome is usually the insulin resistance associated with obesity. Several mechanisms are currently considered to cause insulin resistance, such as abnormal lipid metabolism and ectopic lipid accumulation [2], and mitochondrial dysfunction [3], in addition to inflammation and endoplasmic reticulum stress [4]. Globally, more than 70% of deaths are due to chronic diseases, such as heart disease, stroke, cancer, and diabetes. These diseases account for two-thirds of disease in middle-income countries and are expected to rise to three-quarters by 2030.

Therefore, in addition to classic strategies such as physical activity and caloric consumption reduction, the identification of new molecular targets capable of preventing, or at least reducing, insulin resistance and its metabolic consequences induced mainly by the heavy consumption of fats represents one of the most important public health challenges.

One of the consequences of the Western lifestyle and high-fat diet is non-alcoholic fatty liver disease (NAFLD), which affects about 30% of adults and up to 10% of children in developed countries [5]. NAFLD begins with hepatic triacylglyceride (TAG) accumulation and is defined as the presence of lipid droplets in the cytoplasm of more than 5% of hepatocytes [6]. This disease develops when the rate of hepatic TAG synthesis, as a result of increased fatty acid uptake and esterification to TAG, as well as de novo lipogenesis from carbohydrate and protein metabolism, exceeds the rate of hepatic TAG catabolism through the oxidation of fatty acids or the secretion of TAG in the form of very low-density lipoproteins (VLDL) [6]. One of the major concerns with NAFLD is that hepatic lipid accumulation has been linked to the development of hepatic insulin resistance and T2DM.

NAFLD represents a broad spectrum of histological abnormalities that range from simple steatosis to non-alcoholic steatohepatitis (NASH) and may progress to cirrhosis or even hepatocellular carcinoma. As observed in wild type mice, a high-fat diet (HFD) produces an obesity phenotype similar to human pathology associated with insulin resistance. However, there is no clear installation of fibrosis after long exposure of up to 50 weeks of this type of diet [7]. Some animal models have emerged as a solution for studying the evolution of NAFLD to NASH. One of the most classic animal models of NASH is induced by a diet deficient in methionine and choline. However, this animal model presents a phenotype of body mass loss accompanied by a reduction in glycemia, being, therefore, dissociated from metabolic syndrome. Because of this, it would be more appropriate to use an animal model that best represents the human disease, usually associated with metabolic syndrome [8].

In 2015, Shierwagen and colleagues [9] identified, in the global Apolipoprotein E knockout mouse (ApoE KO) fed a Western diet (high-fat, cholesterol-enriched diet) for 7 weeks, the characteristics inherent to NASH. However, these animals were not characterized with concern to glucose metabolism. These animals had hepatic steatosis, fibrosis, inflammation, obesity and high blood pressure, characterizing an animal model of NASH associated with metabolic syndrome. However, the diet used in this study was supplemented with 1.5% cholesterol, an amount well above the average consumed by humans. Thus, our study aimed to characterize this experimental model of NASH with a Western diet enriched with a lower concentration of cholesterol (0.21%), in addition to analyzing parameters related to glucose metabolism.

## 2. Materials and Methods

### 2.1. Animals

Male ApoE KO mice at 8 weeks of age were obtained from the animal facility at the Institute of Biomedical Science of the University of Sao Paulo. They were maintained in a temperature-controlled room at 22 ± 2 °C with free access to food and water and a light–dark cycle of 12 h. The mice were divided into three groups: regular chow-fed mice (Chow), high-fat fed mice (HFD—D12451, Research Diets, New Brunswick, NJ, USA), and Western diet-fed mice (WD—D12079B, Research Diets, New Brunswick, NJ, USA). The diet composition is described in Table 1. The animals were fed these different diets for 7 weeks before the experiments. All experimental procedures were performed following the “Guidelines for the ethical use of animals in applied etiology studies” and were previously approved by the Ethics Committee on the use of animals at the ICB-USP (CEUA nº 5372180319).

### 2.2. Evaluation of Body Weight and Fat

In awake mice, total body mass and fat mass were analyzed using the LF50 minispec Body Composition Analyzer (Bruker, Billerica, MA, USA).

### 2.3. Glucose Tolerance Test

Awake mice were submitted to an intraperitoneal glucose tolerance test (ipGTT), as previously published [10]. After 6 h of fasting, each mouse received an intraperitoneal injection of a solution of 10% glucose (1 mg/g body weight), and the blood samples were collected from a superficial cut in the tail at the following times 0, 15, 30, 45, 60, 90 and 120 min to determine plasma glucose. Extra plasma was collected at time 0′ to measure basal plasma insulin concentrations by mouse insulin Elisa commercial kit (Mercodia^®^).

### 2.4. Measurement of Liver Enzymes in Plasma

After euthanasia, the blood of the animals was collected and centrifuged (2637 g for 15 min) for plasma separation. Then, the plasmatic concentration of liver enzymes (alanine aminotransferase—ALT and aspartate aminotransferase—AST) was analyzed with the commercial kit LabTest (Minas Gerais, Belo Horizonte, Brazil). The dosage of liver enzymes was performed according to the kit protocol.

### 2.5. Tissue Content of Triglycerides

After 6 h of food restriction, the animals were euthanized, and the tissues removed for analysis of the lipid content. Tissue triglycerides (TAG) were extracted using the Bligh and Dyer method [11] and measured using a TAG reagent according to the manufacturer’s instructions (Bioclin, Belo Horizonte, Minas Gerais, Brazil).

### 2.6. Liver and Plasma Cytokine Measurements

The MYCITOMAG-70K-05 kit was used for simultaneous analysis of IFN-γ, IL-1β, IL-6, MCP-1, and TNF-α (Millipore, Darmstardt, Germany) in liver samples and serum. Liver samples were homogenized in the buffer solution T-PER Tissue Protein Extraction Reagent (Thermo Scientific, Waltham, MA, USA) containing a cocktail of protease and phosphatase inhibitors (Halt Protease and Phosphatase Inhibitor, Thermo Scientific). The samples were centrifuged at 12,000 g for 10 min at 4 °C after homogenization to remove nuclei and cell debris. Protein quantification was performed using the Bradford colorimetric method (kit Bio-Rad Protein Assay, Hercules, CA, USA). The collected blood samples were centrifuged at 2637 g for 15 min to obtain serum after coagulation. The multiplex assays were performed in a multi-user facility of our institution (Laboratório de Análises Especiais-LIM03, University of Sao Paulo School of Medicine), following the manufacturer’s recommendations. The data are expressed as pg/μg of protein for cytokine measurement in hepatic tissue and pg/mL for measurements in serum.

### 2.7. Liver Histology

#### 2.7.1. Oil Red O (ORO) Staining

Oil Red O (ORO) Staining. The tissue-tek initially embedded liver samples (Thermo Scientific, Waltham, MA, USA) were placed in isopropanol alcohol and immediately frozen in liquid nitrogen (N2). Twelve-micron slices were prepared using a cryostat (Microm H560 Thermo Scientific, Waltham, MA, USA). Three slices from different parts of the samples were disposed per slide, and two slides per animal were used. The slides were stained with ORO and Mayer’s hematoxylin. Ten images from each animal were obtained using a microscope with 20× objective magnification. The identification of ORO stained area was performed using the ImageJ program [12].

#### 2.7.2. Picrosirius Red Staining

To evaluate liver morphology, as previously published [13], the liver was fixed in 10% formaldehyde solution for 8 h, in individual cassettes. Subsequently, the fixed samples were kept overnight in 70% alcohol. The samples were then dehydrated through a series of baths in 95% alcohol, 100% alcohol and xylene. Once the samples were dehydrated, the tissue samples were embedded in paraffin at 60 °C. A microtome (Zeiss, Jena, Germany) was used to cut the samples into 5 micron slices. The slices were stained with Picrosirius to identify collagen fibers. Twenty-five images using 20× magnification (3 distinct slices) from 5 animals per group were analyzed. Ten images from each animal were obtained using a microscope with 20× objective magnification using a Nikon Eclipse Ti-U microscope coupled with a Nikon DS-R1 digital camera and NIS-Elements BR 3.1 software. The abundance of collagen deposition in the liver was presented as the mean of the percentage from each animal per group. The identification of picrosirius stained area was performed using the ImageJ program. The image was opened in the program, converted to 8-bit grayscale (*Image → type → 8-bit*). Then the following sequence was performed: *Image → Adjust → Threshold; Analyze → Set measurements.* The results were obtained through *Analyze → Measure* [12]. Area values were used as a result, which corresponded to the amount of collagen in the tissue.

#### 2.7.3. Immunohistochemistry (IHC)

In order to analyze the markers for inflammatory infiltration, the liver samples were fixed in a 10% formaldehyde solution for 8 h, following a dehydration of the samples, and then the samples were embedded in paraffin at 60 °C. Five μm thick tissue sections were cut transversely on a microtome (Zeiss, Jena, Germany), and antigen retrieval was performed by heating (96 °C) the samples cut in sodium citrate buffer. Following that, the sections were washed with PBS, incubated with 3% hydrogen peroxide and treated with 10% goat serum for 1 h at 22 °C in a humidified chamber. Then, histology slides were incubated overnight at 4 °C with the primary rat polyclonal antibody F4-80 (Bio-Rad AbD Serotec, Kidlington, UK) diluted in a 5% BSA solution (1:200). The sections were then incubated with the appropriate secondary antibody (Sigma-Aldrich, San Luis, MO, USA) for 1 h at RT. For the visualization of immune complexes, the vectastain ABC-kit (Vector Laboratories, Newark, CA, USA) was applied for 1 h at RT for signal amplification, and 3,3-diaminobenzidine diluted in PBS containing 0.03% (*v*/*v*) H_2_O_2_ was utilized as the chromogen, yielding the overall brown color. Negative controls were prepared for each antibody by omitting the primary antibody. At least two samples from each animal were independently analyzed by two experienced investigators. Images were acquired with a Nikon DS-R1 digital camera connected to a Nikon Eclipse Ti-U microscope. The immunostained sections were quantified by using the Image Pro Plus software (Media Cybernetics, MD, USA). At least 5 areas per slide were selected and photographed in a blinded manner. The expression of F4/80 was calculated as follows: the total area of liver (specimen) was outlined, and the area occupied by cells expressing F4/80 was quantified using the image analyzer within the reference area. A color for F4/80 was pre-defined and applied to the selected area. The result was expressed as a percentage of positive area in relation to the total area. F4/80 is an active macrophage marker, so an increase in the expression of this marker indicates an increase in the inflammatory process.

### 2.8. Real Time Polymerase Chain Reaction (RT-PCR)

Total RNA from the liver was extracted with Trizol reagent (Thermo Scientific, Waltham, MA, USA) and reverse transcribed into cDNA (High-Capacity cDNA kit, Applied Biosystems, Waltham, MA, USA). Gene expression was evaluated by RT-PCR using Rotor Gene Q (Qiagen, Hilden, Germany) and SYBR Green as the fluorescent dye (Platinum^®^ SYBR^®^ Green qPCR Supermix UDG, Invitrogen, Waltham, MA, USA). The gene expression analysis was carried out using a method previously described [14,15]. The primers used are described in Table 2.

### 2.9. Statistical Analysis

The GraphPad Prism version 9.0^®^ program (GraphPad Software, La Jolla, CA, USA) was used to analyze the data. The D’Agostino and Pearson omnibus normality test was used to determine the distribution of the samples, using the GraphPad Prism software. The samples were analyzed using either a two-way ANOVA followed by the post hoc Bonferroni multiple comparison test or a *Student*-*t* test (*p* < 0.05). The results were expressed as mean ± standard error of mean (mean ± SEM).

## 3. Results

### 3.1. Increased Adiposity, Insulin Resistance and Glucose Intolerance in HFD and WD Fed Mice

**Figure 1:**
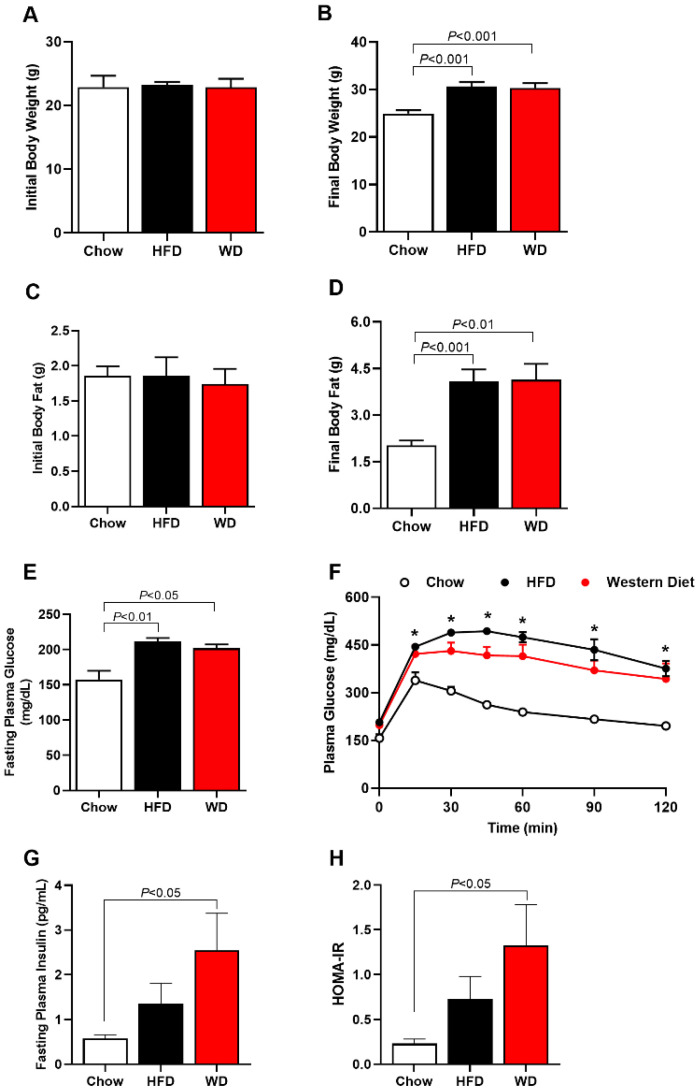
ApoE KO Mice fed an HFD or WD displayed increased body weight, body fat, fasting plasma glucose, plasma insulin and glucose intolerance. Initial body weight (before diet) (**A**); final body weight (after 7 weeks of HFD or WD) (**B**). Initial body fat (before diet) (**C**); final body fat (after 7 weeks of HFD or WD) (**D**); fasting plasma glucose (**E**). Area under curve (AUC) of plasma glucose at different times (**F**). Fasting plasma insulin (**G**). Homeostasis model assessment of insulin resistance (HOMA-IR) (**H**). Data are represented as mean ± SEM (N = 5–9). The statistical differences as indicated by one-way ANOVA were as follows: * *p* < 0.05 represents the difference between Chow (regular diet) vs. HFD; Chow (regular diet) vs. WD.

Whole-body weight was increased in HFD-fed and WD-fed mice after 7 weeks by ~22% and ~20%, respectively, compared with Chow-fed mice (Figure 1A,B). Additionally, whole-body fat was increased in HFD-fed and WD-fed mice after 7 weeks by ~100% and ~105%, respectively, compared with Chow-fed mice (Figure 1C,D). Associated with these increases in body weight and body fat, HFD-fed mice and WD-fed mice displayed an increase in fasting plasma glucose and glucose intolerance compared with Chow-fed mice (Figure 1E,F). HFD-fed mice and WD-fed mice also showed increased fasting plasma insulin compared with Chow-fed mice (Figure 1G), suggesting increased insulin resistance (Figure 1H). There was no difference in these parameters between HFD-fed mice and WD-fed mice.

### 3.2. Increased Plasma Cholesterol, Hepatic Enzymes, and Hepatic Steatosis

**Figure 2:**
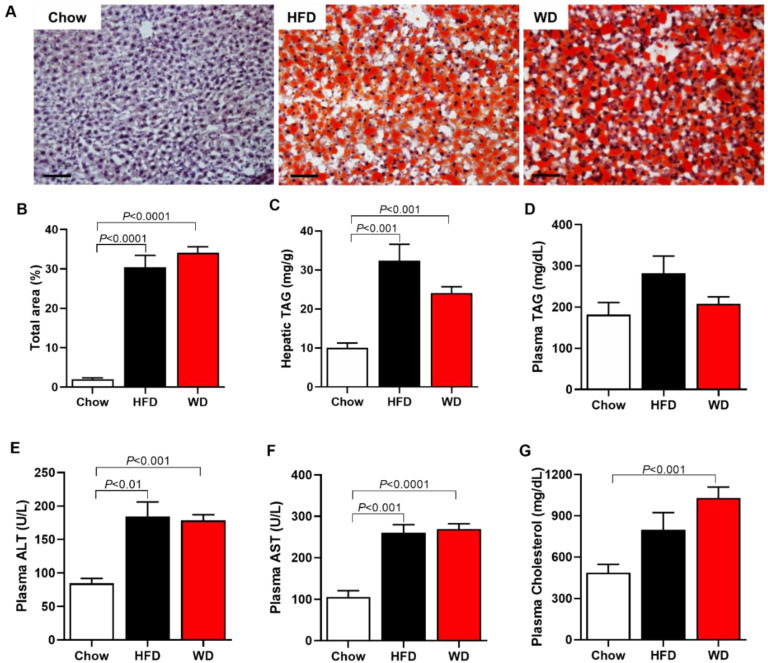
ApoE KO Mice fed an HFD or WD displayed increased liver triglycerides, hepatic enzymes (ALT and AST), plasma cholesterol and lipid droplets. Representative imagens of lipid droplets in the liver, oil red staining (scale of 100 μm, objective 20×) (**A**). Total area of lipid droplet (**B**). Hepatic triglycerides (TAG) (**C**). Plasma triglycerides (TAG) (**D**). Plasma alanine aminotransferase (ALT) (**E**). Plasma aspartate aminotransferase (AST) (**F**). Plasma cholesterol (**G**). Data are represented as mean ± SEM (N = 5–9). The statistical differences as indicated by one-way ANOVA.

WD-fed mice displayed an increase in plasma total cholesterol by 111% compared with Chow-fed mice (Figure 2G). At the same time, there was no difference between the groups in plasma TAG concentration (Figure 2D). Mice fed an HFD or a WD also displayed increased plasma concentration of hepatic enzymes, ALT and AST, when compared with Chow-fed mice (Figure 2E,F). Associated with these data, both HFD-fed and WD-fed mice showed increased hepatic steatosis compared with Chow-fed mice (Figure 2A–C).

### 3.3. Increased Fibrosis and Inflammation in HFD-Fed and WD-Fed Mice

**Figure 3:**
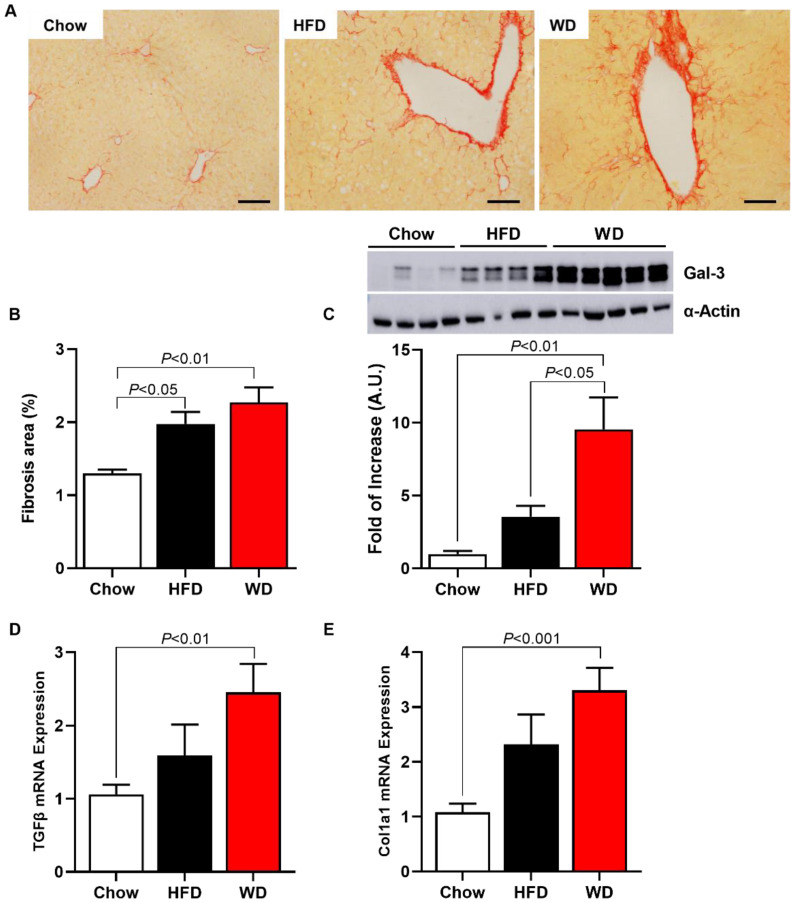
ApoE KO Mice fed an HFD or WD displayed increased hepatic fibrosis. Representative images of collagen deposition, picrosirius staining (scale of 50 μm, objective 40×) (**A**). Area fibrosis (**B**). Protein expression of galectin-3 (representative images—Western blot) (**C**). Pro-fibrogenic gene expression (**D**,**E**). Data are represented as mean ± SEM (N = 5–9). The statistical differences as indicated by one-way ANOVA.

HFD-fed and WD-fed mice displayed increased fibrosis area by 46% and 76%, respectively, compared with Chow-fed mice (Figure 3A,B). Interestingly, a Western blot for Galectin-3 (Gal-3), a protein associated with the fibrosis pathway, showed that WD-fed mice displayed increased Gal-3 expression in the liver by 850% compared with Chow-fed mice and by 170% compared with HFD-fed mice (Figure 3C). There was no significant increase in hepatic Gal-3 expression in HFD-fed mice compared with Chow-fed mice.

**Figure 4:**
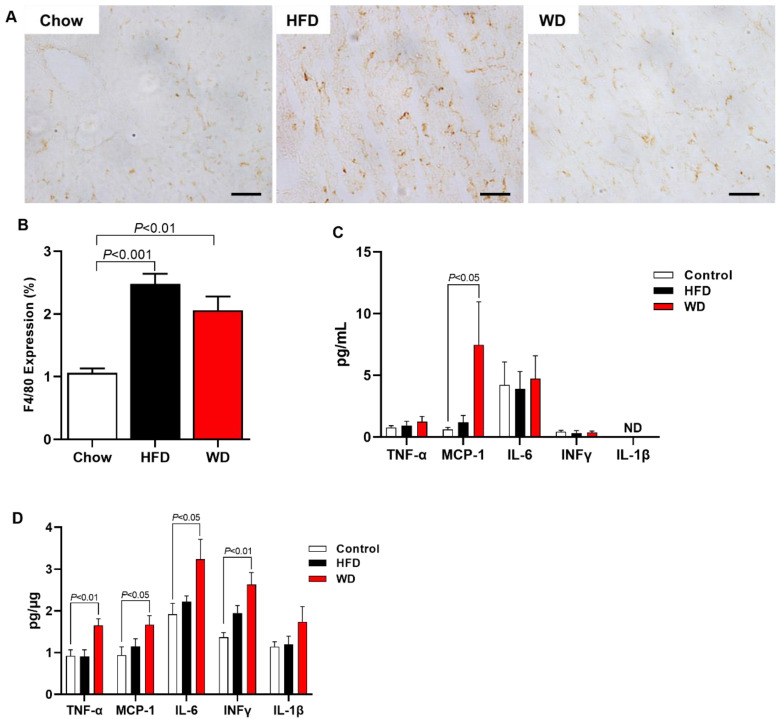
ApoE KO Mice fed an HFD or WD displayed increased hepatic inflammation. Representative images of F4/80 expression (scale of 50 μm, objective 40×) (**A**). Hepatic inflammation represented for F4/80 expression (macrophage activity marker) (**B**). Plasma cytokine level (**C**). Hepatic cytokine level (**D**). Data are represented as mean ± SEM (N = 5–9). The statistical differences as indicated by one-way ANOVA.

Inflammation, another hallmark of NASH, was also measured by histology in the liver and by a multiplex kit in the liver and plasma. HFD-fed mice and WD-fed mice displayed increased F4/80 expression in the liver measured by histology compared with Chow-fed mice (Figure 4A,B). Interestingly, the plasma measurement inflammation marks showed that only MCP-1 was increased in plasma from WD-fed mice compared with Chow-fed mice (Figure 4C). However, WD-fed mice presented increased inflammation in the liver, displaying increased content of TNF-α, MCP-1, IL-6, and INFγ compared with Chow-fed mice (Figure 4D). In contrast, HFD-fed mice did not display increased inflammation.

## 4. Discussion

This study characterized an experimental model of NASH with a cholesterol-enriched diet (Western diet). In this model, an increase in adiposity, plasma cholesterol, insulin resistance and glucose intolerance was observed, in addition to an increase in plasma liver enzymes, steatosis, inflammation and hepatic fibrosis.

ApoE KO mice at 12 months of age have increased serum levels of triglycerides, glucose, and insulin, in addition to hepatic steatosis and increased plasma levels of liver enzymes, demonstrating liver damage [16]. Furthermore, 2-month-old ApoE KO mice fed a high-cholesterol diet (Western diet) showed increases in the same parameters, demonstrating the influence of diet [17].

Studies have shown increased adiposity and hepatic steatosis in ApoE KO mice fed WD for 14 weeks [18]. In addition to body weight gain, an increase was reported in fasting blood glucose, hepatic triglyceride, inflammation, and liver fibrosis in ApoE KO animals fed WD for 7 weeks [9]. Lee et al. [19] also reported increased body weight, fasting glucose, insulin, leptin, cholesterol, and triglyceride in ApoE KO mice fed a WD for 12 weeks. Our study demonstrated similar changes in these parameters in ApoE KO animals fed a WD for 7 weeks. In addition, we observed glucose intolerance in WD-fed mice and a more suitable model for diet-induced NASH when mice were fed a WD compared with HFD-fed mice.

ApoE KO mice fed HFD showed an increase in body weight, plasma cholesterol, steatosis, and liver enzymes (ALT and AST) [20,21]. These data agree with our study, which showed an increase in these parameters in animals fed with HFD or WD, with no significant difference between the diet groups.

In addition, ApoE KO mice fed an HFD showed hepatic inflammation, with an increase in pro-inflammatory cytokine (TNF-α, IL-6) expression and transcription factor NF-kB [21]. Our data demonstrated increased macrophage marker activity (F4/80) in HFD- and WD-fed ApoE KO mice. We also saw an increase in TNF-α, IL-6, and INFγ cytokines, in addition to monocyte chemotactic protein-1 (MCP-1) in WD-fed ApoE KO mice.

Bartelt et al. [22] reported an increase in fasting glucose, plasma insulin, and glucose intolerance in ApoE KO mice fed HFD. In another study, insulin resistance was demonstrated in addition to glucose intolerance in the same animal model [23]. These data are similar to what we showed in our study, such as glucose intolerance and increased plasma insulin, suggesting insulin resistance in HFD-fed and WD-fed ApoE KO mice.

Our study also showed an increase in galectin-3 (Gal-3) protein expression in WD-fed ApoE KO mice. This increase was not seen in HFD-fed ApoE KO mice. The increase in gal-3 was also shown in the aorta of this same animal model fed a WD [24].

Gal-3 is a chimera-like protein belonging to the lectin family [25]. It can stimulate fibroblast proliferation in vitro and is highly expressed in different fibrotic diseases in humans and murine models [26,27]. Furthermore, the differentiation of quiescent fibroblasts into active myofibroblasts capable of secreting extracellular matrix proteins is vital to fibrogenesis. Gal-3 can also act as a cofactor of TGFβ in the differentiation of fibroblasts into active myofibroblasts [28].

In wild-type mice, an HFD produces an obesity phenotype similar to human pathology associated with insulin resistance [29]. However, there is no precise installation of fibrosis after prolonged exposure of up to 50 weeks to this type of diet [7].

Some animal models have emerged as a solution for studying the evolution of NAFLD to NASH. One of the most classic animal models of NASH is that induced by a diet deficient in methionine and choline. However, this animal model presents a phenotype of body mass loss accompanied by a reduction in glycemia, being, therefore, dissociated from metabolic syndrome. Because of this, it would be more appropriate to use an animal model that best represents the human disease, usually associated with metabolic syndrome [8].

According to our results, Western diet-fed ApoE KO mice represent a suitable model of diet-induced NASH. They displayed an accumulation of lipids in the liver, followed by an inflammatory process indicated by the increase in pro-inflammatory cytokines, an active macrophage marker, and hepatic fibrosis. In addition, these mice displayed insulin resistance and glucose intolerance, parameters related to metabolic syndrome, mimicking the human disease. In conclusion, once this rodent NASH model of a relatively short-term development is characterized in this study, it will be possible to use it for future investigations of disease mechanisms and molecular targets for treatment and test experimental and suitable treatments for the disease.

## Figures and Tables

**Table 1:** Diet composition.

	Regular Diet (Chow)	High Fat Diet (HFD)	Western Diet (WD)
Carbohydrate	56%	41%	50%
Fat	3.5%	24%	21%
Protein	19%	24%	19%
Cellulose	5%	-	-
Vitamins and minerals	4.5%	6%	4.2%
Fiber	-	5%	5%
Cholesterol	-	-	0.21%
Calories	3.2 Kcal/g	4.7 Kcal/g	4.7 Kcal/g

**Table 2:** Primer sequences.

Col1a1	sense 5′GCTCCTCTTAGGGGCCACT3′ anti-sense 5′CCACGTCTCACCATTGGGG3′
TGFβ	sense 5′CTCCCGTGGCTTCTAGTGC3′ anti-sense 5′GCCTTAGTTTGGACAGGATCTG3′
B2M	sense 5′CCCCACTGAGACTGATACATACG3′ anti-sense 5′CGATCCCAGTAGACGGTCTTG3′
